# Growth Promotion of Maize Exposed to Arsenic and Mercury with a Consortia of Rhizosphere Bacteria Isolated from Mining Tailings

**DOI:** 10.1007/s00284-025-04393-w

**Published:** 2025-08-06

**Authors:** Daniel Rojas-Solis, Yolanda Magdalena García Rodríguez, John Larsen, Gustavo Santoyo, Roberto Lindig-Cisneros

**Affiliations:** 1 Laboratorio Nacional de Innovación Ecotecnológica para la Sustentabilidad, Instituto de Investigaciones en Ecosistemas y Sustentabilidad, Universidad Nacional Autónoma de México, Morelia, Michoacán Mexico; 2 Laboratorio de Diversidad Genómica, Instituto de Investigaciones Químico-Biológicas, Universidad Michoacana de San Nicolás de Hidalgo, Edifcio A1’, Ciudad Universitaria, 58063 Morelia, Michoacán Mexico

## Abstract

In this study we evaluated plant growth promotion (PGP) traits of bacteria associated with maize (*Zea mays* L.) including *Pseudomonas* sp. (TL36), *Staphylococcus* sp. (TL49), *Gottfriedia* sp. (TL52), and *Bacillus* sp. (TL80), isolated from mining tailings, when exposed to mercury and arsenic. Initially, the response of the bacteria to the heavy metals studied were evaluated in in vitro assays, showing regular-moderate tolerance to arsenic and low tolerance to mercury. Also plant growth promotion traits of the bacteria were studied in in vitro assays in terms of production of indole-3-acetic acid (IAA), phosphate solubilization, siderophores and biofilm when exposed to arsenic and mercury. In addition, production of volatile organic compounds by the four bacteria were measured. In general, bacterial PGP traits were maintained when exposed to arsenic though some differences were observed between bacteria and individual PGP trait. On the other hand, all the PGP traits were strongly reduced when exposed to mercury for all bacteria. Also, the compatibility between the four bacteria was examined in vitro. From the abovementioned results two consortia were established based on the TL36 and TL80 (consortium 1) and, TL49 and TL52 consortium 2, which were tested for plant growth promotion after single or multiple inoculations in maize exposed to arsenic and mercury. Single and multiple inoculation, with consortium 1 with *Pseudomonas* sp. and *Bacillus* sp. increased maize plant growth similarly, while on the other hand, consortium 2 with *Staphylococcus* sp. and *Gottfriedia* sp. had no effect on maize plant growth. Despite the observed moderate tolerance to arsenic of bacteria in consortium 1, the observed plant growth promotion was mitigated in the presence of arsenic.

**Supplementary Information:**

The online version contains supplementary material available at 10.1007/s00284-025-04393-w.

## Introduction

Heavy metal contamination is one of the most relevant problems in contemporary agriculture. Their high toxicity and ability to accumulate in soils and crops represent a serious threat to agricultural production and human health [1]. Mine tailings have been identified as one of the most important sources of pollution. Particularly, abandoned mines could increase the content of heavy metals in surface- and groundwater, sediments, and agricultural soils [2, 3].

Heavy metals are highly toxic compounds that can persist in the soil for a long time, particularly arsenic and mercury, which can also mix with agricultural soil if mining activities are carried out nearby [4]. Mercury is a persistent and mobile element, and its volatility, renders it unstable [5]. Mercury contamination in soil is associated with a toxic effect on humans and ecosystems, causing changes in membrane permeability, changes in the structure of macromolecules, induces oxidative stress and mitochondrial dysfunction, in addition to increasing reactive oxygen species [6].

It is estimated that the total global amount of mercury accumulated in the soil environments range from 200 to 300 mg kg^−1^, so the concern generated by this element is magnified due to its ability to accumulate in different trophic levels in food chains [7]. Arsenic is a metalloid that occurs in the environment in different oxidation states including As (+ 3), As (0), and As (+ 5). It is highly toxic to plants, animals, and humans, causing disturbances by inhibiting the activity of more than 200 enzymes, growth inhibition, alteration of the respiratory and photosynthetic systems, generating the production of reactive oxygen species, among others [8]. Furthermore, it is the twentieth most abundant element in the Earth's crust and can be found in the range ~ 5 mg kg^−1^ and 10 mg kg^−1^ [9].

Contamination with heavy metals in agricultural soils reduces the availability of macronutrients and increases soil acidity, which hinders the establishment of crops, in addition to altering the diversity and structure of microbial communities, their functioning, and essential biochemical traits such as respiration, fixation of nitrogen, and mineralization of nitrogen and phosphorus [10].

Owing to the negative impacts caused by the presence of heavy metals in the soil, remediation strategies have been developed that include physical, chemical, and biological methods. Physical and chemical strategies, although considered appropriate, are difficult to apply and generate very high costs. Furthermore, their effect on the diversity of rhizosphere microorganisms and soil fertility makes their application even more difficult [11]. Although biological methods, or bioremediation processes, are eco-friendly, cost-effective, less labor-intensive, and have an advantage over physical and chemical methods, bioremediation encompasses several techniques that use plants, animals, and microorganisms [12, 13]. Phytoremediation utilizes various types of plants that have the capacity to absorb metals. Through processes such as translocation, accumulation, transport, transformation, and volatilization, these plants reduce the amounts of contaminants present in the environment [14, 15]. Some plant families, such as Brassicaceae, Euphorbiaceae, Asteraceae, Fabaceae, Lamiaceae, Poaceae, and Scrophulariacea, are recognized as excellent accumulators of heavy metals [16]. For example, maize crops, in addition to being used as a staple food source due to their high nutritional value and high yield, have been used as an optimal model plant for phytoremediation due to their rapid growth and high biomass production [17]. Asilian et al. [18] carried out greenhouse trials to evaluate the capacity of maize crops to reduce the amounts of lead (Pb) present in the soil, examined in terms of microbe assisted phytoremediation. Their results showed that the application of surfactants and the inoculation with the endophytic fungus *Piriformospora indica* and the rhizosphere bacterium *Pseudomonas fluorescens* promoted the reduction of Pb, favoring phytoextraction and phytostabilization processes.

Another way to carry out bioremediation is with plant growth-promoting rhizobacteria (PGPR) resistant to heavy metals [19]. These PGPRs contain the genetic material that makes them resistant to the presence of metals by presenting various detoxification mechanisms, including biosorption, trapping, efflux, reduction, precipitation, and complexation [20]. The key plant promotion traits of PGPRs include IAA production, phosphate solubilization, siderophore secretion, biofilm production, and the emission of volatile organic compounds, VOCs [21, 22]. Many bacterial genera has been reported as PGPR agents that also participate in the biodegradation of metals such as: *Acinetobacter, Actinobacteria, Alcaligenes, Arthrobacter, Bacillus, Beijerinckia, Flavobacterium, Methylosinus, Mycobacterium, Mycococcus, Nitrosomonas, Nocardia, Xanthobacter, Phanerochaete, Pseudomonas* and *Serratia* [20, 23, 24].

Pinter and collaborators [25] evaluated the capacity of a consortium with *Bacillus licheniformis, Micrococcus luteus*, and* P. fluorescens* to reduce the toxicity of As(-III) in grapevines (*Vitis vinifera* L. cv. Malbec), finding that plant inoculation with the consortium stimulated the growth of the crop and the yield of the fruits. Additionally, the consortium limited the entry of arsenic into the plant by increasing the defense mechanisms in the crop, reducing the toxic effects.

Interactions between microorganisms and plants play a fundamental role in eliminating contaminating elements in ecosystems. In this context, microbe-assisted phytoremediation, offers an effective alternative to improve and accelerate bioremediation by favoring soil methylation, releasing siderophores, organic acids, and phytohormones in the rhizosphere, and inducing changes in soil pH [26].

Franchi and collaborators determined the phytoextraction of Hg and As through a microbe-assisted phytoremediation process, using *Brassica juncea* and *Lupinus albus* species and microorganisms, adding thiosulfate as a mobilization agent, resulting in phytoaccumulation up to 85% and 45% for As and Hg, respectively.

In our work group, we have isolated, characterized and selected strains from cultures established in mine tailings with PGPR capacity, highlighting the isolates TL36, TL49, TL52 and TL80 [27]. Thus, the objective of this study was to validate growth promotion traits to evaluate the effect of single or multiple inoculations of the TL36–TL80 and TL49–TL52 consortia on maize plants exposed to arsenic and mercury. We hypothesized that the frequency of inoculation directly influences the plant growth promotion effects exerted by these consortia.

## Materials and Methods

### Biological Materials

Previously, we conducted a search for culturable microorganisms obtained from the rhizosphere of maize established in mine tailings located in Tlalpujahua, in the State of Michoacán, México [27]. The Tlalpujahua mining district is characterized by a system of epithermal veins, where extensive gold and silver mining took place. Extraction methods favored the accumulation of heavy metals, particularly mercury and arsenic, which exceeded the permissible levels of these metals in the soil according to NOM-147-SEMARNAT/SSA1-2004.

Microorganisms were collected from maize crops (N 19 48′ 28.8″, O 100 10′ 03.6″; N 19 48′ 26.8″, O 100 10′ 00.2″). Microorganisms were isolated from 0.5 g soil samples diluted in 1 mL of water, which were further diluted and plated on nutritive agar (BD Bioxon, Becto Dickinson de México) culturing medium in Petri dishes that were incubated 24 h at 30 °C.

The isolates *Pseudomonas* sp. TL36, *Staphylococcus* sp. TL49, *Gottfriedia* sp.TL52 and *Bacillus* sp. TL80 were selected according to their tolerance to arsenic and mercury, in addition to their plant growth promotion traits, including high production of siderophores, biofilm, 3-indoleacetic acid (IAA), and phosphorus solubilization [27]. The bacteria were grown at 30 °C for 24 h on NA plates and routinely maintained at 4 °C. The isolates were incubated at 28 °C for 24 h and preserved in 20% glycerol at − 80 °C.

### Molecular Characterization and Phylogenetic Analysis of TL36, TL49, TL52 and TL80

Genomic DNA was isolated from the bacteria stains TL36, TL49, TL52, and TL80, and the 16S ribosomal DNA subunit (rDNA) was amplified using polymerase chain reaction (PCR) with the universal bacterial primers fD1: 5′-CAGAGTTTGATCCTGGCTCAG-3′ and rD1: 5′-AAGGAGGTGATCCAGCC-3′ under previously reported PCR conditions [28]. PCR conditions were as follows: an initial denaturation at 95 °C for 3 min, 30 cycles of denaturation for 1 min at 95 °C, annealing for 1 min at 53 °C, extension for 2 min at 72 °C, and a final extension step at 72 °C for 5 min. PCR amplifications were performed using the TC-142 Thermocycler Techne thermal cycler (Keison Products, Chelmsford, UK) in Go Taq Master Mix tubes (Promega, Madison, WI, USA). The PCR product was further purified, and the 16S rDNA regions of the bacterial isolates were sequenced. Partial 16S rRNA sequences of the strain isolates were compared with available sequences by the BLAST search in the National Centre for Biotechnology Information (NCBI) database (http://www.ncbi.nlm.nih.gov). The best NCBI matches were selected from complete genomes with complete ribosomal RNA gene sequences. Identification was based on the criteria reported by Chun et al. [29].

### Bacterial Growth in the Presence of Heavy Metals

To evaluate the response of *Pseudomonas* sp. TL36, *Staphylococcus* sp. TL49, *Gottfriedia* sp. TL52 and *Bacillus* sp. TL80 to heavy metals, the strains were cultivated for 48 h in different concentrations of Na_2_HAsO_4_, NaAsO_2_ (200, 600, and 1000 mg L^−1^), and HgCl_2_ (50, 75, and 100 mg L^−1^), in LB medium (complemented with the respective concentrations of the various metals). Growth was measured to an optical density (OD) of 590 nm and determined starting from OD = 0.1. The growth of the bacterial cells was evaluated at different time intervals with a UV–Vis spectrophotometer (GENESYS 20).

### Determination of Plant Growth Promotion Traits

The IAA content was determined following the method described by Rojas-Solis et al. [30], with some modifications. Flasks with 25 mL of nutrient broth (5 g peptone and 3 g yeast extract L^−1^) were inoculated, complemented by different metallic salts (Na_2_HAsO_4_, NaAsO_2_, and HgCl_2_) with 4 concentrations (36, 72, 75, and 150 mg L^−1^). The flasks were then placed at 30 °C in a rotary shaker at 150 rpm. The cells were collected through centrifugation at 10.000 × *g* for 15 min, and 2 mL of Salkowski reagent was added to the supernatant. The absorption corresponding to the complex auxin rose was recorded at 540 nm in a UV–Vis spectrophotometer (GENESYS 20). The calibration chart was created using dilutions of a standard solution of IAA using concentrations 0, 2, 4, 6, 8, 10, 15, 20, 30, 40, 50 and 60 μg mL^−1^ (Fluka, Switzerland) and the medium uninoculated with the reagent as the control. The experiments were performed in triplicate.

Siderophore production was evaluated in a chrome azurol S (CAS) agar medium, medium supplemented (or not) with 75 and 150 mg L^−1^ (Na_2_HAsO_4_ and NaAsO_2_) and 36 and 72 mg L^−1^ (HgCl_2_) [30]. The experiments were performed in triplicate.

Phosphate solubilization assay, the Pikovskaya medium [with Ca_3_(PO_4_)_2_ as phosphate source, 5 g L^−1^] was used. The medium supplemented (or not) with 75 and 150 mg L^−1^ (Na_2_HAsO_4_ and NaAsO_2_) and 36 and 72 mg L^−1^ (HgCl_2_) [30]. The experiments were performed in triplicate.

The biofilm formation capacity of the TL36, TL49, TL52 and TL80 was analyzed following the protocol of Wei and Zhang [31]. Strains were cultivated in LB (Merck KGA, Darmstadt, Germany), supplemented (or not) with 75 and 150 mg L^−1^ (Na_2_HAsO_4_ and NaAsO_2_) and 36 and 72 mg L^−1^ (HgCl_2_), to an OD of 1 (*A*_570_) and then diluted (1:1000) with fresh LB broth. A 0.5 mL diluted culture was transferred to an Eppendorf tube. Bacteria were incubated without agitation for 24 h at 30 °C, and the biofilm was quantified in this time. The biofilm was stained with 0.1% (w/v) crystal violet for 15 min at room temperature and then rinsed thoroughly with water to remove unattached cells and residual dye. Ethanol (95%) was used to solubilize the dye that had stained the biofilm cells. The absorbance of the solubilized dye (*A*_570_) was determined using a UV–Vis spectrophotometer (GENESYS 20). The experiments were performed in triplicate in at least two independent sets.

### Analysis of Volatile Organic Compounds

For the analysis of the volatile organic compounds (VOC’s) emitted by the different bacterial isolates, which were grown on NA, the bacteria were incubated at 30 °C for 24 h. The VOC’s were identified through the solid-phase microextraction technique and gas chromatography coupled with mass spectroscopy (SPME–GC–MS) using PDMS/DVB fibers (Supelco, Bellefonte, PA, United States) according to the protocol established by Hernandez-León et al. [28] with some modifications. A DB-23 capillary column (30 m × 0.32 mm × 0.25 μm) was used, with a fluid of 1 mL min^−1^ of helium as a gas carrier. The column was maintained for 1 min at 40 °C and was later programmed to increase by 3 °C min^−1^ until finally reaching 180 °C, where it was maintained for 1 min. The conditions of the detector were as follows: a temperature of the interface of 250 °C, ionization through electric impact at 70 eV, and SCAN mode (40–350 *m*/*z*). The VOC’s were identified by a comparison with data from the mass spectral library (NIST/EPA/NIH, “Chem-Station,” Agilent Technologies Rev. D.04.00 2002). Three independent determinations for each strain were performed.

### Compatibility Assay

Prior bacterial co-inoculation plant experiments, compatibility assays were performed according to Garbeva and De Boer [32] to test for possible antagonism between the isolates TL36, TL49, TL52, and TL80. The isolates were grown in nutrient broth (NB) until reaching an OD of 1. Afterward, a drop of 100 μL (1 × 10^6^ CFU mL^−1^) was deposited in a petri dish of NA, and once dry, it was sealed and stored at 30 °C for 4 days. This treatment helped measure individual bacterial growth. For the compatibility assay, a drop of 100 μL of TL36 was deposited simultaneously in another Petri dish with nutrient agar, and while leaving 1.5 cm between them, a drop of 100 μL of TL80 was placed. This same procedure was carried out for the TL49 and TL52. Once the drops dried, the dishes were sealed and stored at 30 °C for 4 days. All the experiments were performed in triplicate. Finally, 1 cm^2^ was recovered from each plate, and dilutions were performed to determine the quantity of CFU present in each treatment.

### Plant Growth Experiment

The experiment was performed in a greenhouse with maize (*Zea mays* L.) variety Puma grown in plastic pots (12 cm high × 11 cm wide) using a sterile substrate composed of soil from an agricultural field with known history of maize production in Tlalpujahua, Michoacán, México (N 19° 48′ 28.8′′, W 100° 10′ 03.6′′) mixed with sand (1:1 v/v). The substrate was sterilized for 2 h at 120 °C and 15 PSI.

Maize seeds were surface sterilized by shaking in 95% ethanol for 2–3 min followed by soaking in 10% sodium hypochlorite with concomitant shaking. Subsequently the seeds were washed 3–4 × in autoclaved distilled water. Seeds were soaked in PGPR inoculated broth for 3–4 h prior sowing, in 50 mL of bacterial suspension (1 × 10^6^ CFU mL^−1^) composed of the TL36–TL80 (consortium 1) and TL49–TL52 (consortium 2) consortia and shaken for 24 h at 150 rpm at room temperature. For the preparation of the consortia, the isolates were grown individually in nutrient broth until reaching an optical density of 1 (600 nm), using 25 mL of each isolate.

All pots received nutrient solution (Hewitt, 1966) [33], regardless of the treatment, and the water content of the substrate was maintained at 60% w/w. The experiments were performed using different metallic salts [(Na_2_HAsO_4_ and NaAsO_2_ (150 mg kg^−1^) and HgCl_2_ (72 mg kg^−1^)], until reaching the desired concentrations as well as the different consortia in the respective treatments.

The experimental design is shown in Table 1. To perform the experiment, the plants were subjected to single and multiple inoculations. The consortia were adjusted to *A*_600_ = 1.0. For the maize subjected to single inoculation, the consortia were applied at the beginning of the experiment placing 5 mL per pot directly on the substrate, while in the treatments with multiple inoculations the same procedure was followed, only the consortia were applied every week from the beginning to the end of the experiment (five inoculations in total were performed); in the case of the control treatments, plants were left noninoculated. The effects of the inoculations on the root and shoot length, chlorophyll concentration, and total dry weight were determined at 5 weeks of plant growth. The chlorophyll concentration was measured using a portable measuring device SPAD-502Plus (Konica, Minolta. Santiago de Chile). The SPAD-502 Plus instantly measures chlorophyll content, uses LED light sources, which allows measuring the amount of light absorbed by this pigment in the leaves, generating a close approximation of the N content in the plant.

**Table 1:** Experimental design of the evaluation of plant growth promotion in the presence of heavy metals

Metallic salts and concentration	Treatment
	Control plants (without inoculation)
	Plants + metallic salt
	Consortium 1 − single inoculation
	Consortium 1 − multiple inoculation
Na_2_HAsO_4_	Consortium 1 + metallic salt − single inoculation
150 mg kg^−1^	Consortium 1 + metallic salt − multiple inoculation
	Consortium 2 − single inoculation
	Consortium 2 − multiple inoculation
	Consortium 2 + metallic salt − single inoculation
	Consortium 2 + metallic salt − multiple inoculation
	Control plants (without inoculation)
	Plants + metallic salt
	Consortium 1 − single inoculation
	Consortium 1 − multiple inoculation
NaAsO_2_	Consortium 1 + metallic salt − single inoculation
150 mg kg^−1^	Consortium 1 + metallic salt − multiple inoculation
	Consortium 2 − single inoculation
	Consortium 2 − multiple inoculation
	Consortium 2 + metallic salt − single inoculation
	Consortium 2 + metallic salt − multiple inoculation
	Control plants (without inoculation)
	Plants + metallic salt
	Consortium 1 − single inoculation
	Consortium 1 − multiple inoculation
HgCl_2_	Consortium 1 + metallic salt − single inoculation
72 mg kg^−1^	Consortium 1 + metallic salt − multiple inoculation
	Consortium 2 − single inoculation
	Consortium 2 − multiple inoculation
	Consortium 2 + metallic salt − single inoculation
	Consortium 2 + metallic salt − multiple inoculation

Consortium 1 = *Pseudomonas* sp. TL36 + *Bacillus* sp. TL80. Consortium 2 = *Staphylococcus* sp. TL49 + *Gottfriedia* sp.TL52. Each treatment consists of 12 replicates

### Statistical Analysis

The results were analyzed by means of analyses of variance and Duncan’s test for mean comparisons using Statistica 8.0 software. Also, for comparisons of two-mean values, a Student’s *t* test (*P* < 0.05) was carried out. The greenhouse test results were analyzed with a one-way ANOVA for each of the metallic salts examined (Table 1) and post hoc mean comparisons were performed with Duncan’s multiple range test (*P* ≤ 0.05).

## Results

### Molecular Characterization of Isolates TL36, TL49, TL52, and TL80

Molecular sequencing of the 16S rDNA gene of TL36, TL49, TL52, and TL80 was performed. The isolate TL36 showed 97.46% of identity with *Pseudomonas putida* strain ATCC 12633^T^ (NR_114479.1). TL49 exhibited 98.55% of identity with *Staphylococcus saprophyticus* subsp. *saprophyticus* ATCC 15305 = NCTC 7292^T^ (NR_074999.2), while TL52 showed 97.35% of identity with *Gottfriedia acidiceleris* strain AFS033236^T^ (OP986849.1). Finally, TL80 presented 97.49% identity with *Bacillus thuringiensis* strain HD1011^T^ (CP009335.1) (Table 2). The GenBank accession numbers of the ribosomal sequences of *Pseudomonas* sp.TL36, *Staphylococcus* sp. TL49, *Gottfriedia* sp. TL52, and *Bacillus* sp. TL80 are PP949384, PP951676, PP951525 and PP951577, respectively. In addition, the bacteria have been deposited into the strain collection of the National Laboratory for Ecotechnological innovation, Institute for Ecosystem Research and Sustainability, National Autonomous University of Mexico Campus Morelia.

**Table 2:** NCBI BLAST results of the 16S rRNA region sequences of selected bacteria isolated from mining tailings

Strain	GenBank accession number	Amplicon size in base pairs	Best NCBI match	Similarity (%)	GenBank accession number
TL36	PP949384	1492	*Pseudomonas putida* strain ATCC 12633^T^	97.46	NR_114479.1
TL49	PP951676	1305	*Staphylococcus saprophyticus* subsp. *saprophyticus* ATCC 15305 = NCTC 7292^T^	98.55	NR_074999.2
TL52	PP951525	1261	*Gottfriedia acidiceleris* strain AFS033236^T^	97.35	OP986849.1
TL80	PP951577	1306	*Bacillus thuringiensis* strain HD1011^T^	97.49	CP009335.1

Best NCBI matches were selected from complete genomes or Type strains with complete ribosomal RNA gene sequences

### Bacterial Tolerance to Heavy Metals

**Fig. 1:**
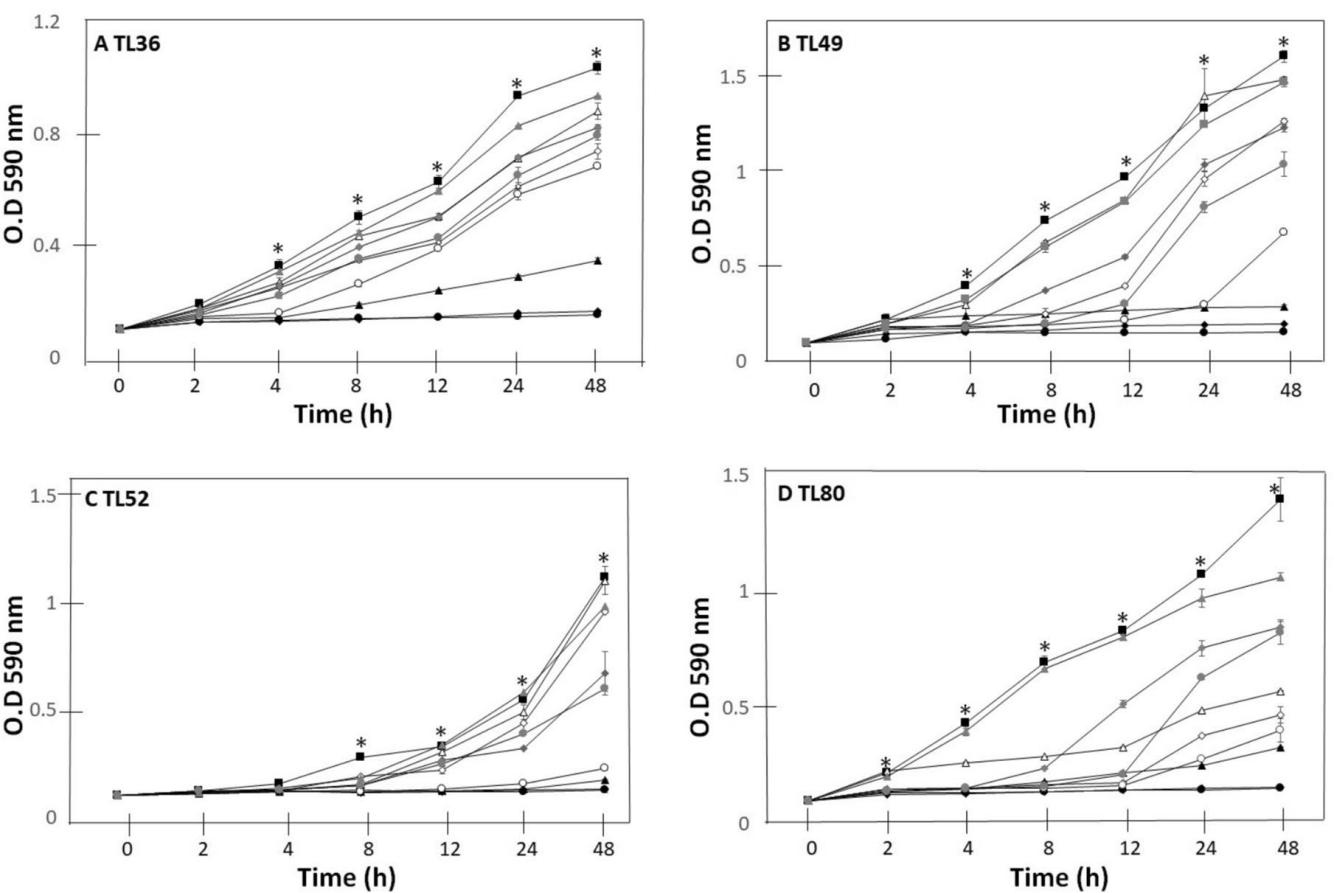
Growth of *Pseudomonas* sp. TL36 (**a**), *Staphylococcus* sp. TL49 (**b**), *Gottfriedia* sp. TL52 (**c**) and *Bacillus* sp. TL80 (**d**), grown in LB media supplemented with heavy metals. The control treatment is represented by closed squares and the then as follows: 200 mg L^−1^ of Na_2_HAsO_4_ by gray triangles; 600 mg L^−1^ of Na_2_HAsO_4_ by gray diamonds; 1000 mg L^−1^ of Na_2_HAsO_4_ by circles gray circles; 200 mg L^−1^ of NaAsO_2_ by white triangles; 600 mg L^−1^ of NaAsO_2_ by white diamonds; 1000 mg L^−1^ of NaAsO_2_ by white circles; 50 mg L^−1^ of HgCl_2_ by black triangle; 75 mg L^−1^ of HgCl_2_ by black diamonds; and 100 mg L.^−1^ of HgCl_2_ by black circles. Statistically significant growth inhibition was observed between treatment and control experiments marked by asterisks; Student’s *t*-test *P* < 0.05

**Fig. 2:**
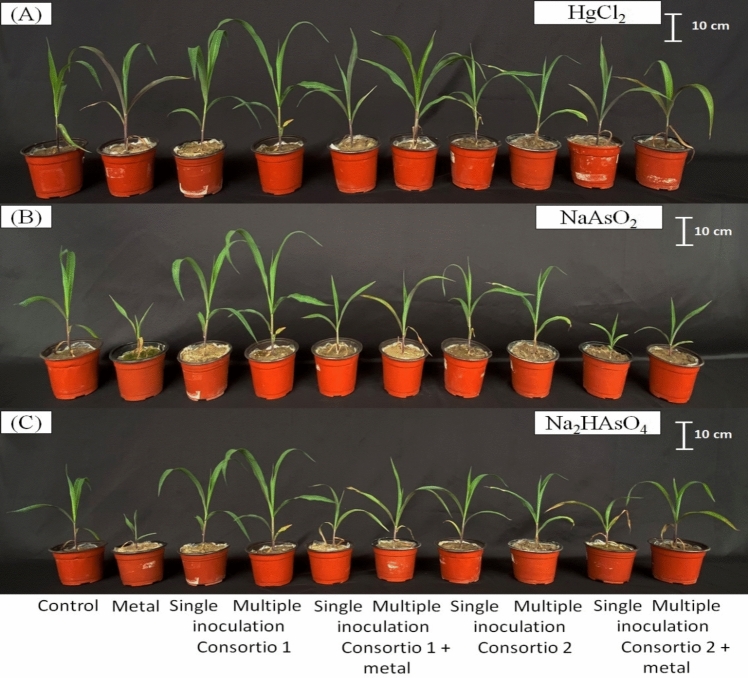
Effect on the development of the aerial part of maize plants (*Z. mays* L.) inoculated with the TL36 + TL80 (consortium 1) and TL49 + TL52 (consortium 2). Panels **a** and **b** represent the plants subjected to concentrations of HgCl_2_ (72 mg kg^−1^) and NaAsO_2_ (150 mg kg^−1^)_,_ respectively. In panel **c**, the plants exposed to Na_2_HAsO_4_ (150 mg kg^−1^) are shown

We evaluated the growth of isolates TL36, TL49, TL52, and TL80 in LB medium supplemented with metallic salts (Fig. 1). Under control conditions without heavy metals, TL36, TL49, TL52, and TL80 reached a growth of 1.035, 1.611, 1.119, and 1.398 (*A*_590_), respectively, at the final evaluation time of 48 h. The addition of NaAsO_2_ was the salt with the lowest negative impact on the growth of the different strains. The strongest inhibition occurred at the maximum concentration tested (1000 mg L^−1^), reducing bacterial growth by 23.38% (TL36), 35.38% (TL49), 41.20% (TL80), and 46.47% (TL52) (percentages obtained at the end of the test). Concerning the effect caused by Na_2_HAsO_4_ on the growth of the isolates, it was determined that for TL36 the addition of 1000 mg L^−1^ reduced growth by 33.91%; for TL49, the percentage of inhibition was 57.79%; while for TL52 and TL80, the reduction in growth caused by this metallic salt was 79.71% and 71.17%, respectively. The higher concentration of HgCl_2_ in the medium exerted greater toxicity on the different isolates, reaching inhibition percentages greater than 85%. When evaluating the minimum concentration tested (75 mg L^−1^), also growth reduction percentages were observed: 66.57% for TL36, 82.12% for TL49, 84.45% for TL52, and 76.39% for TL80 (Fig. 2).

### Bacterial Plant Growth Promotion Traits

When evaluating plant growth promotion traits, the highest IAA production under control conditions was measured for *Bacillus* sp.TL80 with an average value of 74.20 ± 5.99 µg L^−1^. Addition of Na_2_HAsO_4_ to the medium did not significantly affect this activity, while surprisingly, by placing NaAsO_2_ at its lowest dose, the production of this phytohormone significantly increased (101.57 ± 2.61 µg L^−1^) and by doubling the quantities of NaAsO_2_, the effect was reversed, producing only 51.00 ± 6.30 µg L^−1^ of IAA. For *Pseudomonas* sp. TL36, the average value of IAA in control conditions was 73.45 ± 2.56 mg L^−1^. Interestingly, the addition of metallic arsenic salts induced an increase in this activity, with values that ranged from 110.97 to 113.75 µg L^−1^. *Gottfriedia* sp. TL52 was the bacterium that presented the greatest stimulation for IAA production with the addition of Na_2_HAsO_4_ and NaAsO_2_, with the first salt quantities of 125.24 and 116.82 µg L^−1^ being found, while with the second at its lowest dose, 112.21 µg L^−1^ were produced, which contrasts with the highest dose used in which an average value of 69.54 µg L^−1^ was measured. Even so, in all conditions with the presence of metallic arsenic salts, higher values were presented than in the control condition, where a value of 34.20 ± 1.02 µg L^−1^ was presented. For *Staphylococcus* sp. TL49, IAA was only produced in the control treatment, presenting average values of 19.27 ± 0.40 µg L^−1^. It is important to highlight that the addition of HgCl_2_ to TL36, TL52, and TL80 completely abolished the production of this phytohormone (Table 3).

**Table 3:** Summary of plant growth-promoting traits of *Pseudomonas* sp. TL36, *Staphylococcus* sp. TL49, *Gottfriedia* sp. TL52 and *Bacillus* sp. TL80 with different metallic salts and concentrations

Bacteria	Metallic salt/concentration	IAA^†^	Siderophores^††^	Solubilization of phosphates^††^	Biofilm^†††^
	Control	73.45 ± 2.56 a	17.37 ± 0.55 a	18.12 ± 1.47 ab	0.08 ± 0.001 a
*Pseudomonas putida* TL36	75 mg Na_2_HAsO_4_	113.75 ± 5.91 b	8.25 ± 0.47 b	22.62 ± 1.32 c	0.05 ± 0.007 c
	150 mg Na_2_HAsO_4_	103.17 ± 1.57 b	7.12 ± 0.42 b	19.12 ± 0.37 ab	0.05 ± 0.005 c
	75 mg NaAsO_2_	113.16 ± 0.25 b	7 ± 0.91 b	21 ± 0.88 bc	0.08 ± 0.010 a
	150 mg NaAsO_2_	110.97 ± 3.57 b	4.5 ± 0.28 c	17.12 ± 1.53 a	0.05 ± 0.002 c
	36 mg HgCl_2_	ND	2.5 ± 0.35 d	1.87 ± 0.37 d	0.06 ± 0.004 bc
	72 mg HgCl_2_	ND	3 ± 0.35 cd	ND	0.07 ± 0.014 a–c
*Staphylococcus saprophyticus* TL49	Control	19.27 ± 0.40	6.12 ± 0.47 a	14.62 ± 0.31 a	0.08 ± 0 bc
	75 mg Na_2_HAsO_4_	ND	3.75 ± 0.25 b	21 ± 1.17 a	0.10 ± 0.010 ab
	150 mg Na_2_HAsO_4_	ND	3.62 ± 0.23 b	16 ± 1.93 a	0.12 ± 0.002 a
	75 mg NaAsO_2_	ND	ND	17.25 ± 1.36 a	0.10 ± 0.003 ab
	150 mg NaAsO_2_	ND	ND	21.25 ± 4.37 a	0.07 ± 0.002 c
	36 mg HgCl_2_	ND	3.75 ± 0.25 b	ND	0.06 ± 0.001 cd
	72 mg HgCl_2_	ND	4 ± 0 b	ND	0.04 ± 0.003 d
	Control	34.20 ± 1.02 d	4.87 ± 0.31 a	ND	0.18 ± 0.001 a
*Gottfriedia acidecileris* TL52	75 mg Na_2_HAsO_4_	125.24 ± 2.70 a	3 ± 0.45 b	ND	0.11 ± 0.022 b
	150 mg Na_2_HAsO_4_	116.82 ± 3.23 ab	2.5 ± 0.28 b	ND	0.08 ± 0.006 bc
	75 mg NaAsO_2_	112.21 ± 4.19 b	2.62 ± 0.47 b	ND	0.07 ± 0.004 c
	150 mg NaAsO_2_	69.54 ± 2.05 c	2.12 ± 0.12 b	ND	0.15 ± 0.008 a
	36 mg HgCl_2_	ND	3.12 ± 0.42 b	ND	0.05 ± 0.002 c
	72 mg HgCl_2_	ND	ND	ND	0.05 ± 0.003 c
	Control	74.20 ± 5.99 b	4 ± 0.40 a	18.87 ± 1.25 a	0.08 ± 0.005 b
*Bacillus thuringiensis* TL80	75 mg Na_2_HAsO_4_	70.39 ± 2.11 b	2.37 ± 0.80 b	12.5 ± 1.54 b	0.08 ± 0.002 b
	150 mg Na_2_HAsO_4_	69.99 ± 3.81 b	4.25 ± 0.47 a	17.12 ± 1.04 a	0.12 ± 0.023 a
	75 mg NaAsO_2_	101.57 ± 2.61 a	3.31 ± 0.47 ab	19.37 ± 1.24 a	0.08 ± 0.005 b
	150 mg NaAsO_2_	51.00 ± 6.30 c	4 ± 0 a	15.75 ± 1.16 ab	0.09 ± 0.008 ab
	36 mg HgCl_2_	ND	1.87 ± 0.31 b	1.5 ± 0.40 c	0.04 ± 0.003 c
	72 mg HgCl_2_	ND	ND	ND	0.04 ± 0.001 c

Lowercase letters indicate significant differences relative to the control in Duncan’s multiple range tests (*P* < 0.05)

*ND* not determined

^†^IAA production measured in µg L^−1^

^††^Measured as diameter in mm of ring zone surrounding colonies

^†††^Measured as absorbance at 570 nm

Concerning the production of siderophores, the highest production was obtained by the isolate TL36 under the control condition (17.37 mm) compared to different treatments with metal salts that showed a reduced activity ranging from 8.25 mm (75 mg L^−1^ Na_2_HAsO_4_) to 3 mm (72 mg L^−1^ HgCl_2_). The isolates TL49 presented average values of 6.12 ± 0.47 mm. When Na_2_HAsO_4_ was added at both concentrations (75 and 150 mg L^−1^), the size of the halos was significantly reduced with values of 3.75 and 3.62 mm, respectively. The presence of HgCl_2_ gave similar values (3.75 and 4 mm), while the NaAsO_2_ salt eliminated this trait. For its part, *Gottfriedia* sp. had an average value in control conditions of 4.87 ± 0.31 mm. The addition of the different metal salts of arsenic significantly reduced this activity with values in the range from 2.12 (150 mg NaAsO_2_) to 3 (75 mg Na_2_HAsO_4_). When adding HgCl_2_ to the medium, the lowest dose generated a value of 3.12 mm, while the highest dose abolished this activity. On the other hand, *Gottfriedia* sp. had an average value of 4.87 ± 0.31 mm in the control treatment. The addition of the different metallic salts of arsenic significantly reduced this activity with values that were from 2.12 (150 mg L^−1^ NaAsO_2_) to 3 (75 mg L^−1^ Na_2_HAsO_4_). When adding HgCl_2_ to the medium, the lowest dose generated a value of 3.12 mm, while the highest dose abolished this activity. Finally, *Bacillus* sp. was he isolate that showed the least changes in siderophore production between the control condition and the salt treatments, observing that only the presence of 75 mg L^−1^ Na_2_HAsO_4_ and 36 mg L^−1^ HgCl_2_ significantly reduced this activity with values of 2.37 and 1.87 mm, respectively. The higher amount of HgCl_2_ eliminated the production of siderophores (Table 3).

The highest phosphate solubilization was presented for TL36 with values of 22.62 and 19.12 mm (75 and 150 mg L^−1^ Na_2_HAsO_4_), 21 mm (75 mg L^−1^ NaAsO_2_), 18.2 mm (control condition), and 17.12 mm (150 mg L^−1^ NaAsO_2_). Supplementing media with HgCl_2_ significantly reduced this activity. The isolate TL80 presented a behavior like TL36. Under control conditions, the average value was 18.87 ± 1.25 mm. The presence of NaAsO_2_ did not affect phosphate solubilization, finding values of 19.37 and 15.75 mm (75 and 150 mg L^−1^) and this activity was not reduced with the presence of 150 mg L^−1^ Na_2_HAsO_4_ (17.12 mm), whereas only the minimum concentration used of this metal salt significantly decreased this capacity (12.5 mm). Again, the presence of HgCl_2_ strongly decreased the trait evaluated. For TL49, there were no significant differences between the control condition and the treatments with arsenic salts, observing values in a range of 14.62 to 21.25 mm. For this strain, both concentrations of HgCl_2_ abolished this capacity. Interestingly, the isolate TL52 was not able to solubilize phosphate under any of the conditions tested (Table 3).

Lastly, the highest biofilm production was reported for *Gottfriedia* sp. with an average value of 0.18 ± 0.001 (unit) in the control condition. In treatments with metal salts, biofilm production was observed in a range of 0.05 to 0.15 units. For *Bacillus* sp. TL80, the maximum concentration of Na_2_HAsO_4_, resulted in the highest biofilm production of 0.12 (unit). For the rest of the treatments with metal salts, including the control treatment, the values were 0.04 to 0.09 (unit). Biofilm production for TL36 was greater in the control condition and at a concentration of 75 mg L^−1^ NaAsO_2_ with 0.08. For the rest of the conditions, this trait was significantly decreased. For *Staphylococcus* sp. TL49, the average value of the control condition was 0.08 ± 0. The addition of Na_2_HAsO_4_ in both concentrations and at the concentration of 75 mg L^−1^ NaAsO_2_ generated the conditions for the highest biofilm production (0.10 and 0.12 units). In the rest of the treatments, this trait was significantly reduced. It should be noted that for the four strains, the presence of HgCl_2_ largely limited this capacity (Table 3).

### Volatile Organic Compound Production

Overall the volatile blends of TL36, TL49, TL52, and TL80 were quite similar according to chemical groups. It is important to note that volatiles detected in nutrient agar media without the inoculation of bacterial strains were discarded. The most common bacterial volatiles included ketones, alcohols, sulfur compounds, alkanos, aromatic compounds, aldehydes, amines, and esters. Only five compounds were found to be shared among the four isolates (2-butanone, methyl thiolacetate, dimethyl disulfide, 4-methyl-heptane, and nonanal) (Table 4). For *Pseudomonas* sp., nine volatile compounds were recorded. The compounds methyl thiolacetate, 4-methyl-heptane (alkane), and nonanal (Aldehyde) were the most abundant, occupying 40.6%, 14.35%, and 12.97%, respectively (Table 4). For the *Staphylococcus* sp., 10 different compounds were identified. The main VOC produced were the sulfur compounds methyl thiolacetate (36.51%) and dimethyl disulfide (8.26%), in addition to the alkane heptane, 4-methyl (12.19%) (Table 4). On the other hand, nine compounds were recorded by *Gottfriedia* sp., with methyl thiolacetate as the predominant compound with 35.68%, followed by 4-methyl-heptane (21.41%), and nonanal (17.84%). Only these three compounds represented almost 3/4 parts of the total identified compounds (Table 4). Finally, *Bacillus* sp. showed the greatest diversity of recorded compounds. The compound 4-methyl-heptane, was the most abundant, with 37.52%, followed by the sulfur compounds methyl thiolacetate and dimethyl disulfide (24.11% and 10.61%), respectively (Table 4).

**Table 4:** Analysis of volatile organic compounds produced by *Pseudomonas* sp. TL36, *Staphylococcus* sp. TL49, *Gottfriedia* sp. TL52 and *Bacillus* sp.TL80 detected by GC/MS analysis

TL80	TL52	TL49	TL36	*R*_*t*_ (min)	Volatile compounds
%	%	%	%		
7.19	5.04	7.54	4.79	0.94	2-Butanone
ND	3.89	ND	ND	1.07	Dimethyl sulfide
24.11	35.68	36.51	40.66	1.26	Methyl thiolacetate
ND	ND	6.22	ND	1.52	1-(3-Butenyloxy)-hexane
ND	ND	ND	4.76	1.72	Isobutanol
10.61	3.67	8.26	8.15	2.39	Dimethyl disulfide
3.28	ND	ND	ND	3.24	Ethylbenzene
37.52	21.41	12.19	12.97	5.14	4-Methyl-heptane
2.91	ND	ND	ND	5.39	Heptanal
2.03	ND	ND	ND	7.52	Dimethyl trisulfide
ND	ND	4.79	ND	8.12	Octanal
2.89	17.84	7.03	14.35	11.53	Nonanal
ND	5.23	ND	4.09	13.58	2-Methylbutyl acetate
ND	3.60	ND	ND	14.79	5-Methyl-2-furancarboxaldehyde
ND	ND	8.19	6.71	15.24	Decanal
3.01	ND	ND	ND	23.47	*N*, *N*′-bis(1-methylethyl)-1, 3, 5-triazine-2, 4-diamine
ND	ND	ND	3.33	17.46	5-Methylheptan-2-one
2.95	3.64	4.72	ND	34.61	2-Hexadecanone
3.51	ND	4.54	ND	38.59	12-Methyltridecan-2-one

*ND* not determined

### Bacteria Compatibility Assay

**Fig. 3:**
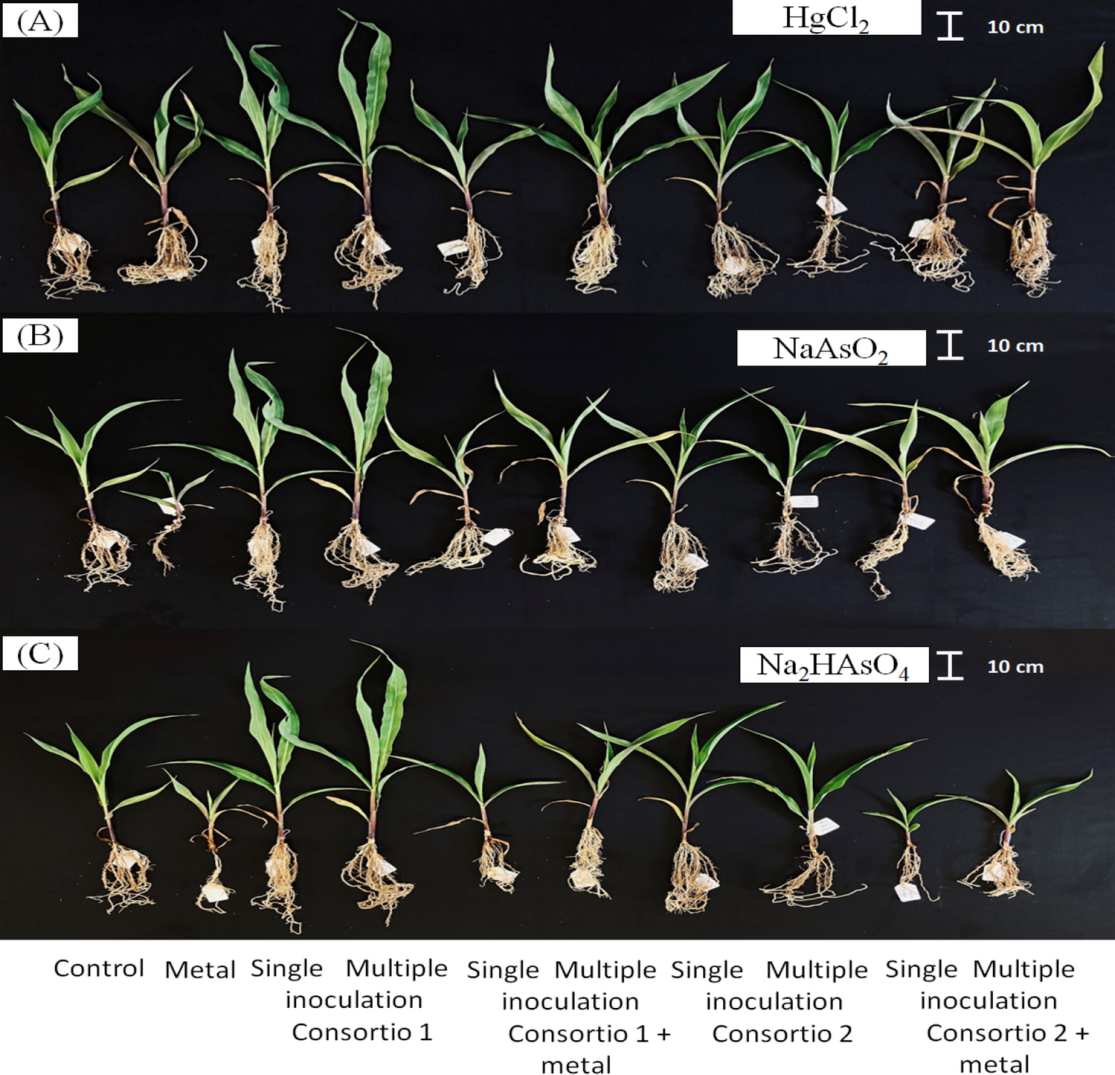
Effect on the development of the aerial part of maize plants (*Z. mays* L.) inoculated with the TL36 + TL80 (consortium 1) and TL49 + TL52 (consortium 2). Panel **a** corresponds to treatments with and HgCl_2_ (72 mg kg^−1^), panel **b** represent the plants subjected to NaAsO_2_ (150 mg kg^−1^), and panel **c**, the plants exposed to Na_2_HAsO_4_ (150 mg kg^−1^) are shown

Before starting the plant assay compatible bacterial consortia were established via antagonist assays. Among the four bacteria TL80 showed the highest growth, reaching an average value of 2.14 × 10^9^ CFU cm^2^, followed by the TL36 (1.77 × 10^9^ CFU cm^−2^), hereafter TL52 with 4.28 × 10^8^ CFU cm^2^, and finally TL49 with 2.17 × 10^8^ CFU cm^−2^. Strains T80 and TL36 reached orders of magnitude of 10^9^, so this was the first consortium that we decided to evaluate. When we evaluated the growth resulting from the interaction of both strains, TL80 reached an average value of 1.20 × 10^9^ CFU cm^−2^, and TL36 reduced its growth to 6.27 × 10^8^ CFU cm^−2^. Despite this, the growth resulting from the interaction of both strains did not show significant differences to their respective individual growth (Table 4). On the other hand, isolates TL52 and TL49 showed growth with orders of magnitude of 10^8^ and were the next consortium that we evaluated. The growth resulting from the interaction did not show significant differences in TL52 (1.27 × 10^8^ CFU cm^−2^) and TL49 (1.27 × 10^8^ CFU cm^−2^) (Supplementary Table 1; Fig. 3).

### Plant Assay

**Fig. 4:**
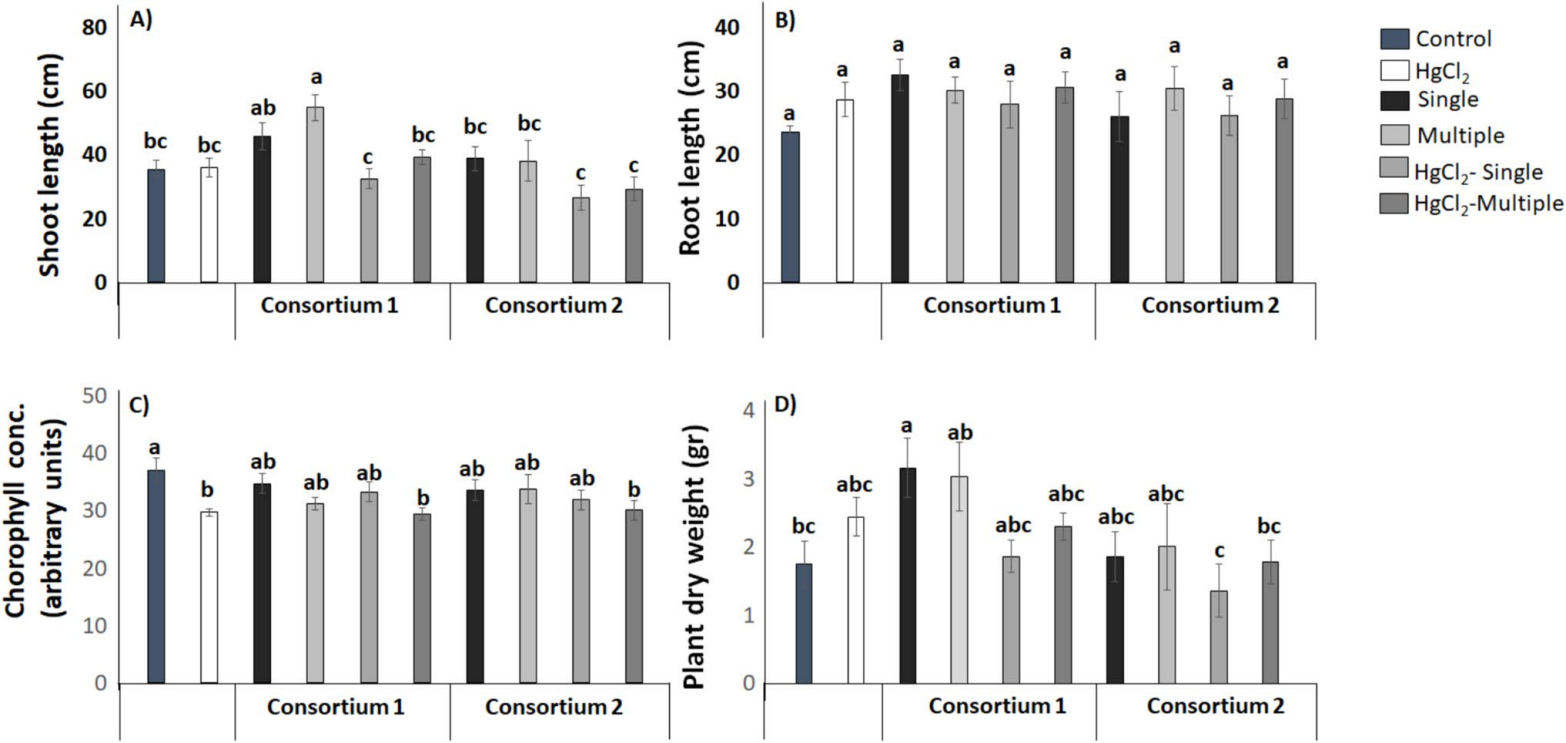
Effect of co-inoculation with consortium 1 (TL36 + TL80) and consortium 2 (TL49 + TL52) on plant growth under different treatments of HgCl_2_ using 72 mg kg.^−1^. Graphics show the shoot length (**a**), root length (**b**), chlorophyll concentration (**c**), and total dry weight (**d**) of maize plants (*Z. mays* L.). The bars represent the mean ± SE. Letters indicate significant differences in the mean values, estimated according to Duncan’s multiple range test (*P* < 0.05)

72 mg kg^−1^ HgCl_2_ to the soil (Fig. 4). In the plant assay with HgCl_2_ consortium 1 (*Pseudomonas* sp. TL36 + *Bacillus* sp. TL80) increased shoot length, when inoculated on multiple occasions without the presence of HgCl_2_, compared to noninoculated plants. The same effect was observed for plant dry weight, but here with single inoculation. For the parameters root length and chlorophyll concentration, no significant differences were found with single or multiple inoculation of this consortium. Consortium 2 (*Staphylococcus* sp.TL49 and *Gottfriedia* sp. TL52) had no effect on any of the plant growth parameters measured.

**Fig. 5:**
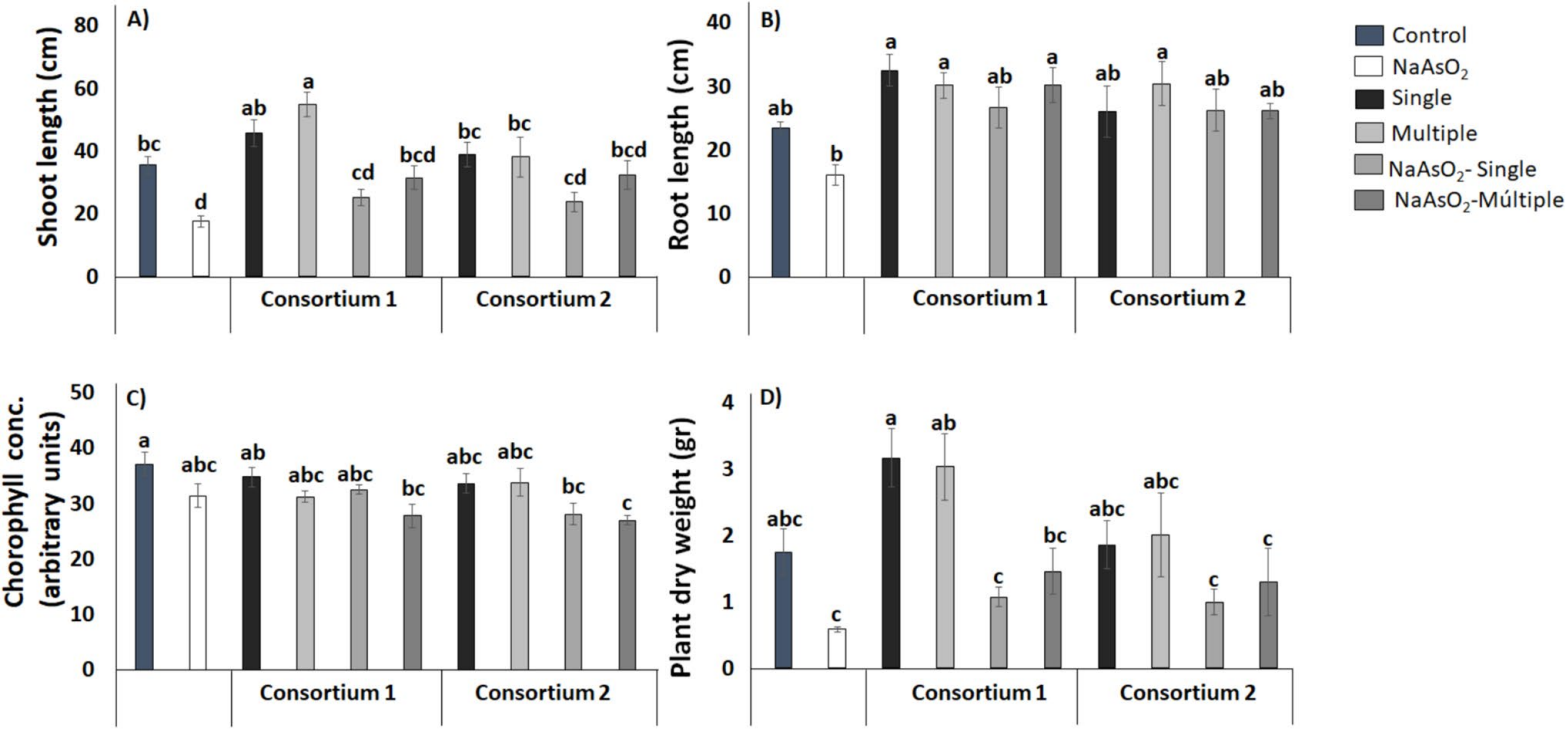
Effect of co-inoculation with consortium 1 (TL36 + TL80) and consortium 2 (TL49 + TL52) on plant growth under different treatments of NaAsO_2_ using 150 mg kg.^−1^. Graphics show the shoot length (**a**), root length (**b**), chlorophyll concentration (**c**), and total dry weight (**d**) of maize plants (*Z. mays* L.). The bars represent the mean ± SE. Letters indicate significant differences in the mean values, estimated according to Duncan’s multiple range test (*P* < 0.05)

In the plant assay with NaAsO_2_ single and multiple inoculation with consortium 1 increased the shoot length compared with the noninoculated control, whereas the root length was only increased with multiple inoculations. Similarly, multiple inoculations of consortium 2 restored root length growth as compared to uninoculated plants. Finally, the chlorophyll concentration and plant dry weight were unaffected by the both bacterial consortia (Fig. 5).

**Fig. 6:**
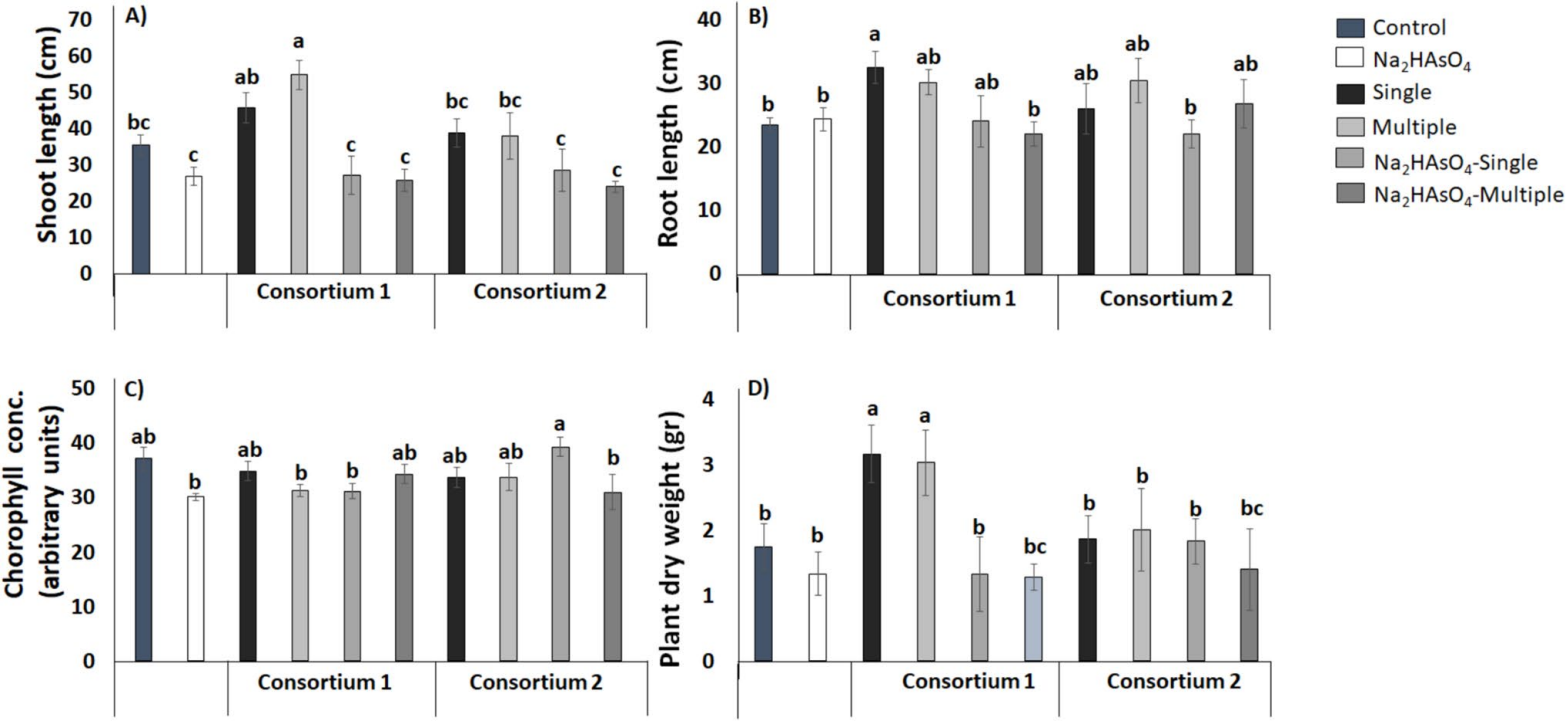
Effect of co-inoculation with consortium 1 (TL36 + TL80) and consortium 2 (TL49 + TL52) on plant growth under different treatments of Na_2_HAsO_4_ using 150 mg kg.^−1^. Graphics show the shoot length (**a**), root length (**b**), chlorophyll concentration (**c**), and total dry weight (**d**) of maize plants (*Z. mays* L.). The bars represent the mean ± SE. Letters indicate significant differences in the mean values, estimated according to Duncan’s multiple range test (*P* < 0.05)

Finally, we evaluated the effect of consortia 1 and 2 by performing single and multiple inoculations in greenhouse trials in the presence of 150 mg kg^−1^ Na_2_HAsO_4_ (Fig. 6).

In the plant assay with Na_2_HAsO_4_, shoot length was increased after either single or multiple inoculations with consortium 1, whereas increased root length was observed after single inoculation with consortium 1, as compared to noninoculated plants. Interestingly, single, and multiple inoculations of consortium 1 increased the plant dry weight when compared to the control.

Inoculation with consortium 2, had no effect on the plant parameters measured, except for a minor increase in the chlorophyll concentration in the plants with single inoculation subjected to Na_2_HAsO_4,_ when compared with the noninoculated control.

## Discussion

Here we validated growth promotion traits (IAA, solubilization of phosphate siderophores, biofilm and volatile organic compounds) of the consortia TL36 + TL80 and TL49 + TL52 under greenhouse conditions in maize plants with single or multiple inoculations subjected to mercury and arsenic.

In a previous study, these isolates had been identified taxonomically by their fatty acid profiles according to the MIDI Sherlock system, obtaining that strain TL36 corresponded to *Herbaspirillum huttiense*, TL49 to *Klebsiella oxytoca*, TL52 and TL80 to *Rhizobium radiobacter*, and *P. putida*, respectively. Nevertheless, one of the limitations of this technique is that the differences between the fatty acid profiles often lack sufficient contrast to accurately establish the identity of the bacteria at the species level [34]. Therefore, in the present study, the isolates were identified molecularly with 16S rDNA primers resulting in the following taxonomic identification: TL36, *Pseudomonas* sp.; TL49, *Staphylococcus* sp.; TL52, *Gottfriedia* sp.; and TL80, *Bacillus* sp. It is important to highlight *Gottfriedia*, members of this genus were transferred from the polyphyletic genus *Bacillus*, so species belonging to the genus *Gottfriedia* form a monophyletic clade in phylogenetic trees based on the concatenated sequences from large protein data sets that differentiate this clade from other genera of the Bacillaceae family [35].

Because tolerance to heavy metals is one of the key factors allowing microorganisms to exert a growth promotion effect in soils subject to these conditions, we evaluated tolerance to Na_2_HAsO_4_, NaAsO_2_, and HgCl_2_. The four isolates exhibited significant tolerance to metallic arsenic salts, attributed to the development of reduction, oxidation, and methylation mechanisms by many PGPR, facilitating arsenic transformation [36]. However, high susceptibility to the presence of mercury was observed, which was expected since this element is extremely toxic due to its high affinity towards sulfhydryl and thioester protein groups, causing the inactivation of these biomolecules, making it more difficult to avoid its toxic effect [37, 38].

Although the capacity of *Pseudomonas* and *Bacillus* as PGPR and bioremediation agents is broad, studies with the genera *Staphylococcus* and *Gottfriedia* are limited. Chhetri et al. [39] highlight the ability of *Gottfriedia endophyticus* RG28T to produce IAA (40.5 µg mL^−1^), showing that *Staphylococcus succinus* H3 exhibited resistance to 4 mM Cu^2+^ and 5 mM Mg^2+^, suggesting an excellent absorption capacity for these metals [40].

Furthermore, evaluating the performance of PGP traits under stress conditions is crucial for establishing relationships between growth promotion and microbe-assisted phytoremediation.

Our finding that the production of IAA by *Staphylococcus* sp. TL49 was completely inhibited by the addition of metal salts is in agreement Khiangte and Lalfakzuala [41], showing that Cu and Fe severely affected the capacity of 17 bacterial isolates to produce IAA including that of *Staphylococcus pasteuri*. However, in contrast, in our study, arsenic increased the production of IAA by *Pseudomonas* sp. (TL36) and *Bacillus* sp. (TL80), which is in accordance to Abou-Shanab et al. [42] who found that a rhizospheric soil spiked with arsenic induced the production of IAA by the bacterial community, coinciding with growth promotion of *Pteris vittata* L.

On the other hand, the release of exudates such as siderophores produced by PGPRs improves the capacity of plant species to carry out bioremediation processes. Mesa et al. [43] found that a consortium with *Ensifer adhaerens* 91R and *Rhizobium herbae* 32E improved the accumulation of arsenic in *Betula celtiberica* leaves, making the phytroextraction processes more efficient. The production of siderophores is a mechanism that different microorganisms have developed to deal with the toxicity of heavy metals, since these molecules can bind to various metals, reducing their concentration and toxic effect in the environment [44]. In our study, metal salt addition significantly impacted siderophore excretion by the isolates. However, *Pseudomonas* sp. TL36 was the isolate that produced the greatest production of siderophores and less affected by the different conditions evaluated.

This finding aligns with Hesse et al. [45], who observed that siderophore producing bacteria (e.g. *Pseudomonas aeruginosa*) were less affected when exposed to metals.

Phosphate-solubilizing bacteria play a key role in increasing plant growth as potential suppliers of soluble phosphate, in addition to showing great potential for the remediation of soils contaminated with heavy metals. The mechanisms via microorganism-mediated P solubilization takes place include enzymatic hydrolysis, dissolution by organic acids, and the release of H_2_S that reacts with iron phosphate to generate ferrous sulfate and soluble phosphate, in addition to the decomposition of plant residues into humic and fulvic acids [46, 47]. In addition, P solubilizing bacteria are capable of immobilizing metals through biosorption, direct absorption of metals, and the release of PO_4_^3−^ to form heavy metal precipitates, reducing the amount of metal salts in the soil [48]. The ability to solubilize phosphate was observed in all our strains except for TL52, and overall this PGP trait was unaffected by the metal salts evaluated, suggesting that P solubilization represent a mechanism supporting growth promotion effects also in heavy metal polluted soils.

Finally, biofilm production is another PGP trait that allows bioremediation processes to be carried out. When we quantified biofilm production in the four bacterial isolates (TL36, TL49, TL52, and TL80), even though the presence of metal salts minimized its production, in all cases, this capacity remained present. Bacteria capable of producing biofilm can grow efficiently under stress conditions, including the presence of heavy metals, using the biofilm generated as a protective layer. This protection is given by the exopolysaccharides that make up the biofilm. In addition, the biofilm produced participates directly in the immobilization and subsequent sequestration of heavy metals, limiting their exposure to the environment [49, 50].

Volatile organic compounds (VOCs) are small molecules (< 300 Da), formed by up to two functional groups, and could diffuse easily. These VOCs are induced by biological factors, interactions, or environmental signals [51]. When identifying the VOCs emitted by our isolates, the shared and most abundant compounds were 2-butanone, methyl thiolacetate, dimethyl disulfide, heptane, 4-methyl, and nonanal. The effects of plant growth promotion and biocontrol have been reported for some of these compounds. Compounds like 2, 3-butanediol emitted by *Bacillus subtilis* GB03 and volatile dimethyl sulfide emitted by *Bacillus* sp. B85 has been shown to promote plant growth in *Arabidopsis thaliana* (L.) Heynh [52, 53]. Chen et al. [54] determined that the nematicide effect exerted by the bacterium *Bacillus aryabhattai* MCCC 1K02966 on *Meloidogyne incognita* was due to the VOCs emitted by these bacteria, mainly pentane, 1-butanol, methyl thioacetate, and dimethyl disulfide, finding that the main compound responsible for this activity was methyl thioacetate. In addition, within the working group, we have found that the nonanal compound produced by *P. fluorescens* UM270 participates in promoting the growth of *Z. mays* L. plants exposed to mercury and arsenic [30].

Finally, bacterial consortia were formed using our four strains. The objective of consortium formation was to promote additive and/or synergistic complementary PGP traits compared to that of single stain inoculum. From the compatibility tests two consortia were established: consortium 1 with *Pseudomonas* sp.TL36 and *Bacillus* sp. TL80 and consortium 2 with *Staphylococcus* sp. TL49 + *Gottfriedia* sp. TL52 (consortium 2). In the maize plant assay with single and multiple bacterial inoculations the strongest plant growth promotion was observed when plants were inoculated with consortium 1 on multiple occasions without being exposed to mercury or arsenic. A greater number of inoculations ensures the persistence of the microorganisms at a high concentration, so the bacterial PGP traits are occurring continuously. In addition, the isolates of consortium 1 *Pseudomonas* sp. (TL36) and *Bacillus* sp. (TL80) showed the highest values of the traits. The evaluated PGP and additive action of both strains exert this prominent promotional effect. Olanrewaju and Babalola [55], showed that inoculation of maize plants with the consortium *P. putida* A18 and *B. subtilis* A1 significantly improved the growth and development of maize plants, determining that the mechanisms responsible for such effects were ammonia production, phosphate solubilization, production of IAA, ACC deaminase activity, and siderophore production.

In the present study, when the plants were exposed to HgCl_2_, neither of the two consortia managed to restore the growth parameters. Mercury is an element that, in contact with plants, exerts a metabolic and physiological disorder, altering mitochondrial activity, modifying the lipids of the biomembrane, generating cell permeability, directly affecting photosynthesis, the water balance of plants, and inhibiting growth [56].

Finally, when the plants were exposed to Na_2_HAsO_4_, inoculation with consortium 2 resulted in increased chlorophyll content compared to the noninoculated plants exposed to this metal. Arsenic is a potential toxic element for plants, and high levels of this metalloid disturb the water relations of plants, promote the generation of reactive oxygen species (ROS), and induce an oxidative burst in plants [57]. Additionally, symptoms of arsenic phytotoxicity include leaf rot and shrinkage, followed by root coloration and difficulty in shoot development [58]. In our study, the plants that were most affected were those grown in the presence of NaAsO_2_, which was expected since As (+ 3) is 10 times more toxic than As (+ 5) [59]. Even so, inoculating plants on multiple occasions with consortium 1 stimulated root development as compared to noninoculated plants. These plant growth promotion and restoration effects on some parameters in plants exposed to mercury and arsenic could be attributed to the synergistic effect of the production of IAA, siderophores, phosphate solubilization, and biofilm, in addition to the production of VOCs such as 2-butanone, methyl thiolacetate, dimethyl disulfide, heptane, 4-methyl and nonanal.

## Conclusions

The results of this work demonstrate that the *Pseudomonas* sp. (TL36), *Staphylococcus* sp. (TL49, *Gottfriedia* sp. (TL52) and *Bacillus* sp. (TL80) showed moderate and limited tolerance to arsenic and mercury, respectively. In addition, all four bacteria possessed PGP traits that potentially contribute to promoting plant growth. Multiple and single inoculations of *Pseudomonas* sp. (TL36) + *Bacillus* sp. (TL80) (consortium 1) and *Staphylococcus* sp. (TL49) and *Gottfriedia* sp. (TL52) (consortium 2) in maize plants allowed us to determine that consortium 1 gave the best results when inoculated multiple times, in uncontaminated soils. This suggests that the combination of *Pseudomonas* sp. (TL36) + *Bacillus* sp. (TL80) represents an excellent bioinoculant option for promoting maize plant growth. As part of future perspectives, further studies in greenhouse and field settings are necessary to evaluate if these bacteria alone and/or in consortia can eliminate, translocate, and/or transform heavy metals.

## Supplementary Information

Below is the link to the electronic supplementary material.

Supplementary file1 (DOCX 13 kb)

## Data Availability

Data will be made available on request.
