# Supplementary material for: Growth Promotion of Maize Exposed to Arsenic and Mercury with a Consortia of Rhizosphere Bacteria Isolated from Mining Tailings
Source: Curr Microbiol. 2025 Aug 6;82(9):438. doi: 10.1007/s00284-025-04393-w (PMC12325397; doi:10.1007/s00284-025-04393-w)
Supplement: Supplementary file 1 — Supplementary file1 (DOCX 13 kb) [file 284_2025_4393_MOESM1_ESM.docx]

**Supplementary table 1.** Bacterial growth determination from single and mixed cultures of *Pseudomonas putida* TL36, *Staphylococcus saprophyticus* TL49, *Gottfriedia acidiceleris* TL52 and *Bacillus* *thuringiensis* TL80.

|  | **Individual (Bacterial CFU cm^-2^)** | **Mix (Bacterial CFU cm^-2^)** |
| --- | --- | --- |
| *P. putida* TL36 | 1.77x10^9^ ± 3.21x10^8^ a | 6.27x10^8^ ± 1.62x10^8^ b |
| *B. thuringiensis* TL80 | 2.14x10^9^ ± 2.22x10^8^ a | 1.20x10^9^ ± 5.00x10^8^ ab |
| *S. saprophyticus* TL49 | 2.17x10^8^ ± 3.18x10^7^ bc | 1.27x10^8^ ± 2.83x10^7^ bc |
| *G. acidiceleris* TL52 | 4.28x10^8^ ± 6.24x10^7^ a | 3.22x10^8^ ± 1.04x10^7^ ab |

To establish that TL36-TL80 and TL49-TL52 strains do not antagonize each other and can coexist and work as a consortium, the growth of the bacteria in nutrient agar medium was evaluated. The growth of the strains individually and in interaction was determined 4 days after the start of the trail. The presence of equal letters denotes that there are no significant differences comparing the growth between both strains individually and as a mixture in Duncan’s multiple range tests (*p* < 0.05).
